# BMI1 regulates multiple myeloma-associated macrophage’s pro-myeloma functions

**DOI:** 10.1038/s41419-021-03748-y

**Published:** 2021-05-15

**Authors:** Danfeng Zhang, Jingcao Huang, Fangfang Wang, Hong Ding, Yushan Cui, Yan Yang, Juan Xu, Hongmei Luo, Yuhan Gao, Ling Pan, Yu Wu, Yuping Gong, Liping Xie, Zhigang Liu, Ying Qu, Li Zhang, Weiping Liu, Wenyan Zhang, Sha Zhao, Qing Yi, Ting Niu, Yuhuan Zheng

**Affiliations:** 1 Department of Hematology, West China Hospital, Sichuan University, Chengdu, China; 2 Department of Hematology, The First Affiliated Hospital of Zhengzhou University, Zhengzhou, China; 3 Department of Pathology, West China Hospital, Sichuan University, Chengdu, China; 4 Center for Translational Research in Hematologic Malignancies, Houston Methodist Cancer Center/Houston Methodist Research Institute, Houston Methodist, Houston, TX USA

**Keywords:** Cancer microenvironment, Myeloma

## Abstract

Multiple myeloma (MM) is an aggressive malignancy characterized by terminally differentiated plasma cells accumulation in the bone marrow (BM). MM BM exhibits elevated MΦs (macrophages) numbers relative to healthy BM. Current evidence indicates that MM-MΦs (MM-associated macrophages) have pro-myeloma functions, and BM MM-MΦs numbers negatively correlate with patient survival. Here, we found that BMI1, a polycomb-group protein, modulates the pro-myeloma functions of MM-MΦs, which expressed higher BMI1 levels relative to normal MΦs. In the MM tumor microenvironment, hedgehog signaling in MΦs was activated by MM-derived sonic hedgehog, and BMI1 transcription subsequently activated by c-Myc. Relative to wild-type MM-MΦs, BMI1-KO (BMI1 knockout) MM-MΦs from BM cells of BMI1-KO mice exhibited reduced proliferation and suppressed expression of angiogenic factors. Additionally, BMI1-KO MM-MΦs lost their ability to protect MM cells from chemotherapy-induced cell death. In vivo analysis showed that relative to wild-type MM-MΦs, BMI1-KO MM-MΦs lost their pro-myeloma effects. Together, our data show that BMI1 mediates the pro-myeloma functions of MM-MΦs.

## Introduction

Multiple myeloma (MM) is an incurable malignancy characterized by accumulation of terminally differentiated plasma cells in the bone marrow (BM). The close interaction between MM cells and the BM microenvironment is essential for MM development, progression and prognosis^1,2^. Macrophages (MΦs) are important and abundant cellular components of MM BM microenvironment. As in other malignancies, in which tumor associated macrophages (TAMs) are “educated” by the tumor microenvironment to acquire cancer promoting functions^3,4^, MM-associated macrophages (MM-MΦs) are influenced by the MM BM microenvironment acquiring a pro-myeloma phenotype. MM-MΦs exhibit greater proliferation, resulting in elevated MM-MΦs levels in MM tumor bed^5^. Additionally, relative to regular MΦs, MM-MΦs confer MM cells with higher protection from chemotherapeutic agents^6–9^. Past studies indicate that MM-MΦs promote angiogenesis in MM BM^10^. However, the mechanisms underlying MM-MΦs’ pro-myeloma differentiation remain unclear. TPL2/MAP3K8 kinase is reported to promote transformation from MΦs to MM-MΦs^11,12^. Aberrant lipid accumulation and metabolism have been reported to influence MM-MΦ differentiation and activation^13^. In this study, we find that BMI1, a member of polycomb-group proteins^14^, regulates MM-MΦs. Relative to normal MΦs, BMI1 expression is elevated in MM-MΦs. We find that BMI1-knockout (BMI1-KO) MΦs do not acquire pro-myeloma functions even within the MM tumor bed. To the best of our knowledge, this is the first study showing BMI1 involvement in MM-MΦs.

## Results

### Characterization of MM-MΦs in a murine myeloma model

**Fig. 1:**
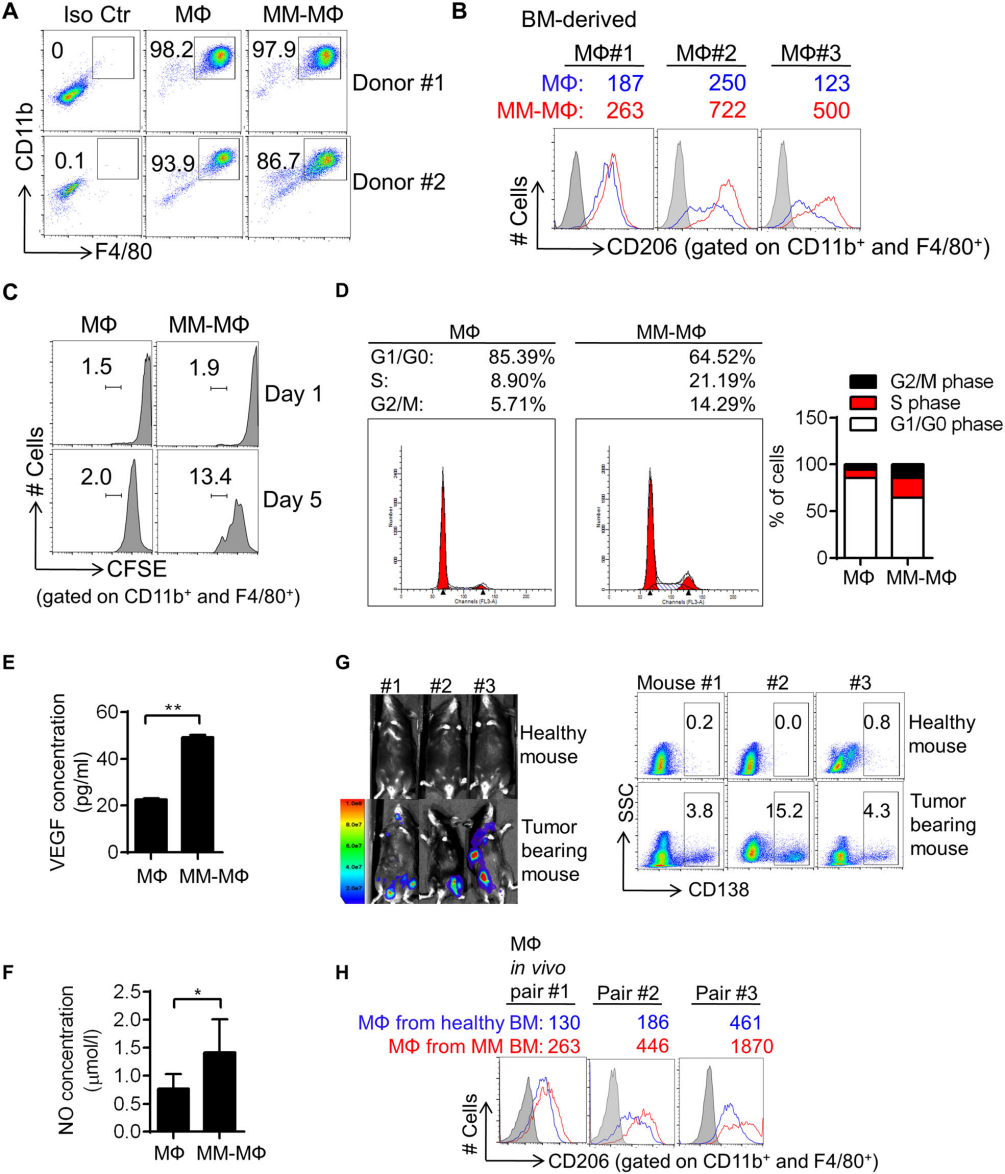
Characterization of MM-MΦs in a murine myeloma model. **A** Flow cytometry showed murine BM-derived MΦs and MM-MΦs were positive of F4/80 and CD11b staining. **B** Mean fluorescent intensity (MFI) of flow cytometry indicated in vitro cultured MM-MΦs expressed higher CD206 than MΦs. **C** In CFSE proliferation assay, MM-MΦs showed higher cell proliferation capacity than MΦs. **D** Cell cycle analysis showed that MM-MΦs had more S and G2/M population, relative to MΦs. **E** ELISA assay detected the concentration of VEGF in culture supernatant of murine BM-derived MФs and MM-MФs. Statistical significance was determined by two-tailed Student *t*-test between MФs and MM-MФs, **P < 0.01. **F** Culture supernatant of murine MM-MФs had higher nitric oxide concentration, relative to MΦs. Statistical significance was determined by two-tailed Student *t*-test between MФs and MM-MФs, *P < 0.05. **G** 5 T murine myeloma model was used for identifying in vivo MM-MΦ features, IVIS photos (left) and CD138 staining (right panel) of BM cells illustrated myeloma tumor burden. Results from 3 representative mice from 7 injected mice are shown. **H** MΦs from myeloma-bearing mice expressed higher CD206 than those from health mice.

To investigate the interplay between MΦ and MM cells, we used the 5 T murine myeloma model^5,15^. One advantage of murine model is that all components in MM BM are syngeneic. The other advantage is that we could use transgenic mice to generate MΦs with target gene modified. Thus, we first characterized features of MM-MΦs vs normal MΦs in vitro and in vivo using the MM murine model. Murine BM-derived MΦs and MM-MΦs were generated in vitro as described in methods section. MΦs and MM-MΦs purity were examined by double-staining for CD11b and F4/80 (Fig. 1A). High CD206 expression was used as a TAM marker^16^. This analysis showed MM-MΦs had higher CD206 levels relative to normal MΦs (Fig. 1B). Using cultured human cells, we have previously shown that MM-MΦs are more proliferative relative to normal MΦs^5^. In murine cells, CFSE (carboxyfluorescein succinimidyl ester) cell proliferation assays revealed greater MM-MΦs proliferation relative to MΦs (Fig. 1C). Cell cycle analysis confirmed that MM-MΦs had a more active cell cycle, as revealed by a higher S and G2/M population relative to normal MΦs (Fig. 1D). Additionally, we found MM-MΦs had a more active protein synthesis than normal MΦs (Supplementary Fig. 1), which was attributable to higher cell proliferation. Furthermore, we found that that MM-MΦs expressed and secreted more angiogenic factors, like VEGF (vascular endothelial growth factor) and NO (nitric oxide)^17^ (Fig. 1E, F). Finally, we generated a 5 T murine myeloma model (Fig. 1G) and analyzed MΦ phenotypes in vivo. Relative to MΦs isolated from the BM of healthy mice, MM-MΦs from tumor bearing mice BM had elevated CD206 levels (Fig. 1H). These MM-MΦ features were used as readouts in downstream analyses aiming to elucidate the molecular factors modulating MM-MΦs.

### BMI1 overexpression in MM-MΦs

**Fig. 2:**
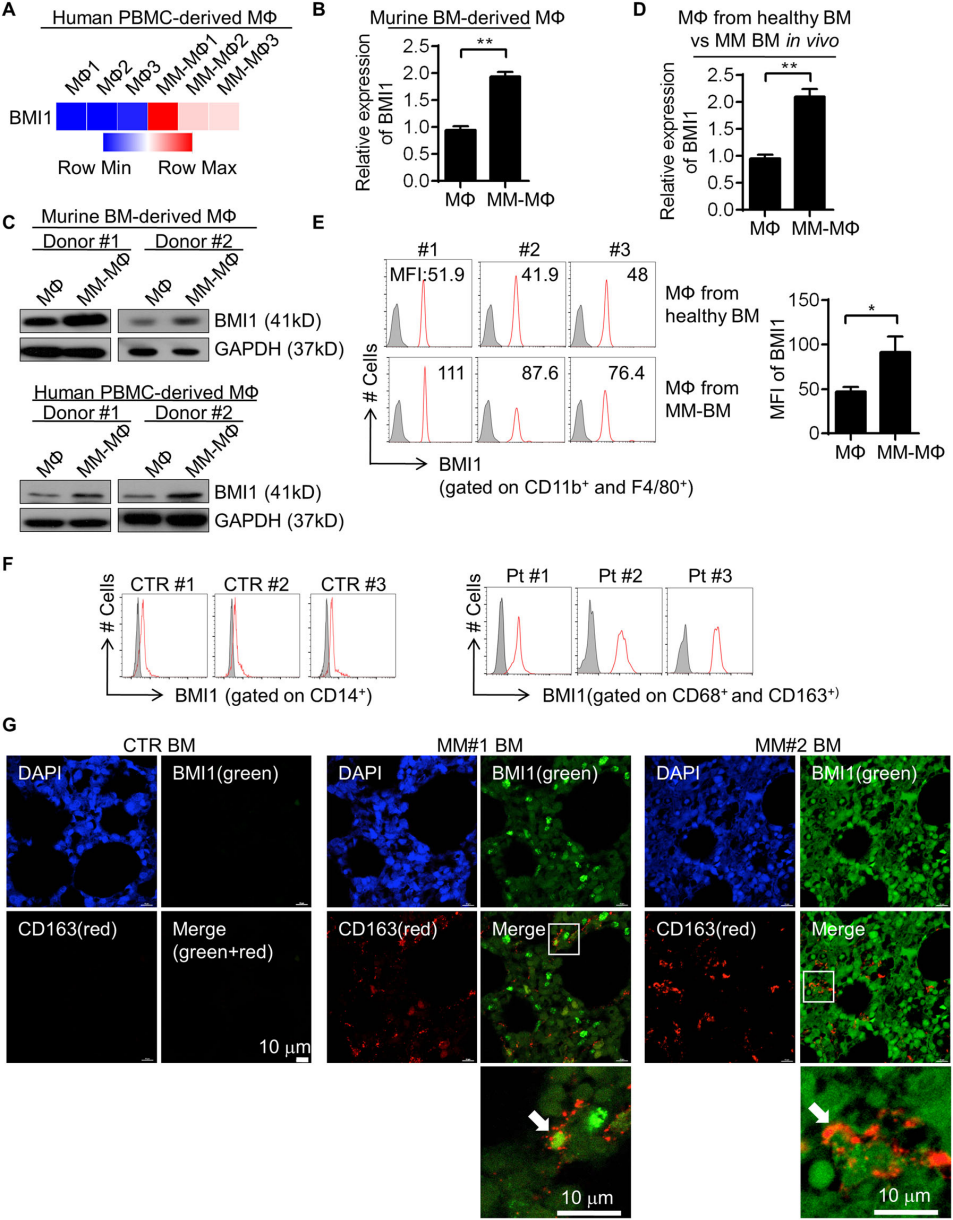
BMI1 overexpression in MM-MΦs. **A** In human peripheral blood monocytes derived MΦs and MM-MΦs, transcriptional microarray analysis showed higher expression of BMI1 in MM-MΦs. **B** In murine bone marrow derived MΦ and MM-MΦ, RT-qPCR showed relative higher BMI1 mRNA expression in MM-MΦs. Statistical significance was determined by two-tailed Student *t*-test between MФs and MM-MФs, **P < 0.01. **C** Western blot showed in both murine BM-derived and human PBMC-derived in vitro culture systems, MM-MΦs had higher BMI1 protein levels. **D** MM-MΦs from the 5T myeloma mice BM expressed higher BMI1 mRNA than MΦs from the healthy mice BM. **E** Left, flow cytometry histograms showed higher BMI1 levels in MΦs from myeloma mice BM. Right panel, statistic result of the mean fluorescence intensity (MFI) of BMI1 in MΦ and MM-MΦ. Statistical significance was determined by two-tailed Student *t*-test between MФs and MM-MФs, *P < 0.05. **F** Left, flow histograms showed the expression of BMI1 in BM CD14^+^ monocytes from 3 of 5 noncancer patients tested. Right, flow histograms showed the expression of BMI1 in BM MΦs from 3 of 5 MM patients tested. **G** Immunofluorescent staining of BM sections from 1 of 3 noncaner control donors and 2 of 5 MM patients showed more MΦs in the MM BM with higher BMI1 expression.

In our previous gene expression profiling of human peripheral blood monocyte (PBMC) derived MΦs and MM-MΦs (Supplementary table 1), we noticed a significant upregulation of BMI1 expression in MM-MΦs (Fig. 2A). RT-qPCR analysis revealed elevated BMI1 levels in murine MM-MΦs as well (Fig. 2B). Similar results were obtained from western blot analysis of murine BM-derived and human PBMC-derived MM-MΦs (Fig. 2C). Next, we used a 5 T murine myeloma model to confirm BMI1 upregulation in MM-MΦs in vivo. We then isolated MM-MΦs from the BM of tumor bearing mice, and normal MΦs from the BM of healthy mice. RT-qPCR and flow cytometry analyses revealed elevated BMI1 levels in MM-MΦs relative to normal BM MΦs (Fig. 2D, E). At last, we examined BMI1 expression in MM patients’ BM MΦs. In noncancer control patients, as previously reported^8^, there were few CD68^+^ MΦs in the BM (data not shown), which were not sufficient to determine BMI1 expression. Flow cytometry showed in control BM, BMI1 expression of CD14^+^ monocytes were quite low. BM MΦs from MM patients revealed relative higher BMI1 expression (Fig. 2F). To validate BMI1 overexpression in patient MM-MΦs, we analyzed BMI1 expression in BM sections from noncancer donors and MM patients using immunofluorescence staining and observed that relative to control BM, MM BM exhibited more MΦs with higher BMI1 levels (Fig. 2G).

### Hedgehog-Myc axis regulates BMI1 overexpression in MM-MΦs

**Fig. 3:**
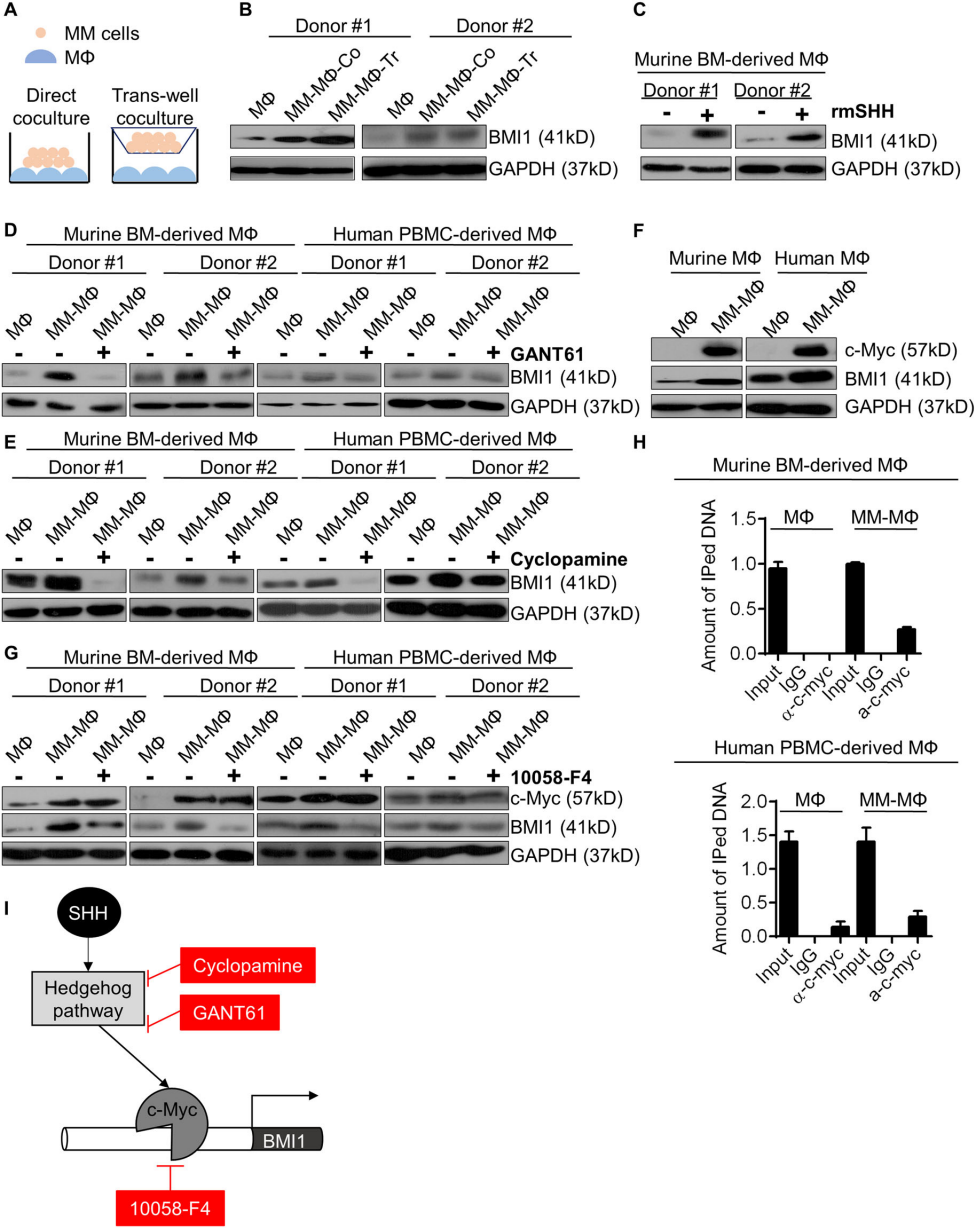
Hedgehog-Myc axis regulates BMI1 overexpression in MM-MΦs. **A** Illustration of direct and trans-well co-culture of MΦs and MM cells. **B** Western blot showed in both direct and trans-well co-culture methods, the expression of BMI1 was elevated in MM-MΦs, relative to MΦs. **C** Western blot showed that protein levels of BMI1 were increased in MΦs upon rmSHH treatment. **D** Western blot showed that the GLI1/GLI2 transcriptional activity inhibitor GANT61 suppressed expression of BMI1 in both murine BM-derived and human PBMC-derived MM-MΦs. **E** Western blot showed that the Smo antagonist Cyclopamine reduced expression of BMI1 in both murine BM-derived and human PBMC-derived MM-MΦs. **F** Western blot showed c-Myc expression was increased in both murine and human derived MM-MΦs. **G** Western blot showed that the c-Myc inhibitor 10058-F4 reduced expression of BMI1 in both murine and human MM-MΦs. **H** ChIP-qPCR showed the relative occupancy of c-Myc and IgG control to BMI1 promoter region in MΦs and MM-MΦs derived from mouse and human. **I** Schematic of Hedgehog-c-Myc axis regulates BMI1 expression in MM-MΦs.

To explore mechanisms of BMI1 upregulation in MM-MΦs, we co-cultured MM cells directly with MΦs or indirectly in trans-wells and observed BMI1 upregulation in both conditions (Fig. 3A, B), suggesting that BMI1 expression in MM-MΦs is modulated by myeloma-derived soluble factors. We therefore focused on soluble factors that are capable of modulating BMI1 expression and are highly expressed in MM BM microenvironment. Myeloma cells have been reported as the main source of the Hedgehog signaling ligand, sonic hedgehog (SHH), in the BM^18^. Hedgehog has been previously reported to modulate BMI1 expression in normal and cancer contexts^19–21^. Thus, we hypothesized that MM-MΦ BMI1 upregulation was modulated by Hedgehog signaling via SHH secretion by myeloma cells. To test this possibility, we treated murine BM-derived MΦs with recombinant mouse SHH (rmSHH) and observed a significant increase in BMI1 levels upon rmSHH treatment (Fig. 3C). Since IL-6 was an important cytokine secreted by TAMs^22^, we also tested whether BMI1 could be regulated by autocrine IL-6. Murine BM-derived MΦs was treated with recombinant mouse IL-6 (rmIL-6). BMI1 level did not change upon rmIL-6 treatment (Supplementary Fig. 2A). Next, we treated MM-MΦs with GANT61 (a GLI1/GLI2 inhibitor) and cylopamine (a Smo antagonist) and observed that both Hedgehog signaling inhibitors attenuated MM co-culture-induced BMI1 elevation (Fig. 3D, E). Hedgehog signaling inhibition also suppressed MM-MΦs proliferation and angiogenic NO levels in culture supernatant (Supplementary Fig. 2B, C).

Given BMI1 mRNA upregulation in MM-MΦ (Fig. 2B), we hypothesized that Hedgehog signaling promoted BMI1 transcription. Analysis of the BMI1 promoter region did not uncover GLI1 or GLI2 binding motifs. However, a binding motif for c-Myc, a downstream effector of Hedgehog signaling^23^ was found. C-Myc has been implicated as a modulator of MΦ polarization into TAM^24^ and is upregulated in MM-MΦs^5^. Expectedly, we observed elevated c-Myc levels in mouse and human MM-MΦs (Fig. 3F). Treating MM-MΦs with 10058-F4, a c-Myc inhibitor, suppressed BMI1 expression and reduced NO concentration in MM-MΦ culture supernatant ((Fig. 3G, Supplementary Fig. 2C). To confirm the elevated c-Myc expression in MM-MΦs was regulated by Hedgehog signaling, we treated MM-MΦs with GANT61 and found suppressed expression of c-Myc upon GANT61 treatment (Supplementary Fig. 2D). We then enforced c-Myc expression in human monocytic leukemia cell line THP-1 via lentiviral infection and stimulated the cells to differentiate into macrophage with PMA (Phorbol-12-myristate-13-acetate)^25^. The suppressive effect of GNAT61 on c-Myc was partially rescued by c-Myc overexpression (Supplementary Fig. 2E). Next, we used ChIP-qPCR analysis to test if c-Myc occupies the BMI1 promoter region in mouse and human MΦ/MM-MΦ cells. Relative to normal MΦs, MM-MΦs exhibited enhanced c-Myc binding to the BMI1 promoter (Fig. 3H). Together, these data suggested that the Hedgehog-c-Myc axis modulates BMI1 expression in MM-MΦs (Fig. 3I). MM BM microenvironment is reported to have high levels of Hedgehog ligands^18,26^, including SHH, which activates Hedgehog signaling in MΦs in MM tumor bed. Thus, Hedgehog signaling activation resulted in c-Myc overexpression, which in turn promoted BMI1 expression.

### BMI1 regulates MM-MΦ proliferation

**Fig. 4:**
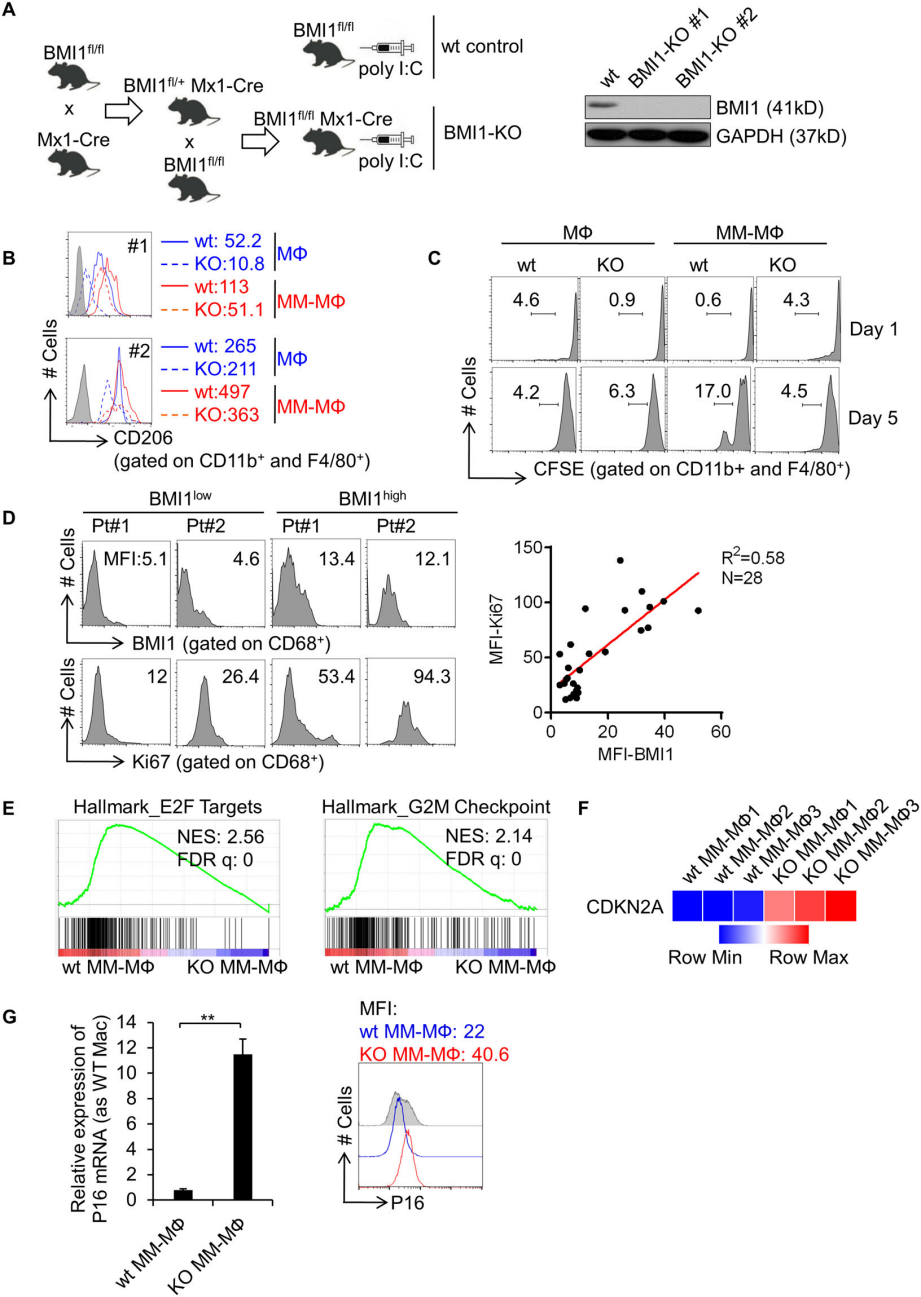
BMI1 regulates MM-MΦs proliferation. **A** Left schematic shows the cross-breedings performed to generate BMI1^fl/fl^Mx1-Cre mice and inducible Cre-mediated disruption of BMI1 in BMI1^fl/fl^Mx1-Cre mice after poly I:C administration. BMI1^fl/fl^ mice received poly I:C were designated as wild-type (wt) controls. Western blot showed the disruption of BMI1 protein in MΦs from BMI1-KO mice after poly I:C administration (right panel). **B** Flow cytometry analyzed the expression of CD206 on the surface of cultured MΦs and MM-MΦs from wt control or BMI1-KO mice. MFI of CD206 indicated for each sample. **C** CFSE cell proliferation assay indicated the knockout of BMI1 in MM-MΦs impaired their proliferation capacity. **D** Flow cytometry analyzed BMI1 and Ki67 expression of MΦs from BM aspirates of 28 MM patients. Left, histograms of BMI1 and Ki67 expression of 2 representative patients with relative lower or higher BMI1 expression. Right panel, the co-expression pattern of BMI1 and Ki67 was showed in dot plots. The correlation was analyzed by linear regression with R square calculated. **E** The RNA-seq data of murine BM-derived MM-MΦs from wt and BMI1-KO mice were analyzed with GSEA, showing enrichment of Hallmark E2F targets and G2M checkpoint pathways in wt MM-MΦs. **F** Heatmap illustrated the RNA-seq data of CDKN2A, showing significant upregulation of CDKN2A in BMI1-KO MM-MΦs. **G** RT-qPCR showed the mRNA expression of P16 was significantly higher in BMI1-KO MM-MΦs, relative to wt MM-MΦs (left column). Flow cytometry showed the protein level of P16 was increased in BMI1-KO MM-MΦs (right panel). **p < 0.01 (Student’s *t*-test, comparing 2 samples, error bars are SD).

To investigate BMI1 functions in MM-MΦs, we generated an inducible BMI1-knockout mouse strain (BMI1^fl/fl^ Mx1-Cre) (Fig. 4A). BMI1^fl/fl^ mice receiving poly I:C were considered the wild-type (wt) control group, while BMI1^fl/fl^ Mx1-Cre mice receiving poly I:C formed the BMI1-knockout (BMI1-KO) group. BMI1-knockout efficiency was verified using western blot analysis of cell lysates from wt or BMI1-KO mice derived BM MΦs. Next, we generated MΦs and MM-MΦs from wt or BMI1-KO mice BM cells. Flow cytometry analysis showed that relative to wt BM-derived MΦs, MΦs and MM-MΦs derived from BMI1-KO cells expressed similar levels of F4/80 and CD11b (Supplementary Fig. 3A), indicating that BMI1-KO MΦs expressed characteristic MΦ markers. Relative to BMI1-KO MΦs, BMI1-KO MM-MΦs exhibited elevated CD206 levels. However, CD206 elevation was not as high as in wt MM-MΦs (Fig. 4B), implicating BMI1 involved in the differentiation from MΦs to MM-MΦs. Additionally, BMI1-KO MM-MΦs exhibited higher population with M1 macrophage markers, MHC-II, than wt MM-MΦs^27^ (Supplementary Fig. 3B).

As discussed earlier, MM-MΦs were significantly more proliferative than normal MΦs, which exhibited limited cell division as terminal differentiated cells. The proliferative potential of MM-MΦs may be critical as it contributes to increased MM-MΦs numbers in the tumor bed. Cell proliferation assays revealed impaired BMI1-KO MM-MΦs proliferation (Fig. 4C). Next, analysis of MM-MΦs from 28 MM patients revealed that the MM-MΦs with high BMI1 levels also had high Ki67 indexes, an indicator of elevated cell proliferation. Linear regression analysis showed positive correlation between MM-MΦs BMI1 and Ki67 expression levels (R^2^ = 0.58) (Fig. 4D), suggesting an association between elevated BMI1 expression and increased macrophage proliferation in MM patients.

Next, we evaluated gene expression profiles in wt MM-MΦ vs BMI-KO MM-MΦ. Pathway enrichment analysis revealed that the Hallmark E2F targets and G2M checkpoint pathways^28,29^ were impaired in BMI1-KO MM-MΦs (Fig. 4E), suggesting their impairment as the molecular basis for the reduced proliferation seen in BMI1-KO MM-MΦs. Our gene expression analyses revealed CDKN2A expression to be significantly upregulated in BMI1-KO MM-MΦs (Fig. 4F). CDKN2A has been reported as a BMI1 target gene. The CDKN2A-encoded p16 protein inhibits the cell cycle by repressing cyclin D binding to Cdk4/6^30^. Our data revealed p16 mRNA and protein upregulation in BMI1-KO MM-MΦs (Fig. 4G). Together, our data suggest that BMI1 is required for MM-MΦs proliferation and that BMI1 downstream factors like CDKN2A (p16), may modulate MM-MΦ cell cycle.

### BMI1 regulates pro-angiogenic function of MM-MΦs

**Fig. 5:**
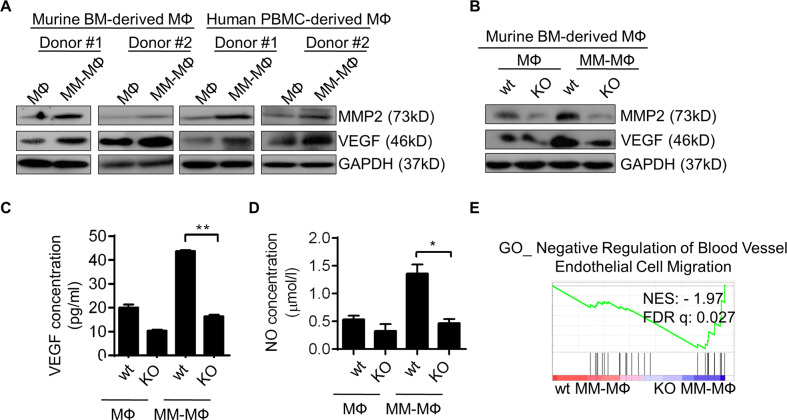
BMI1 regulates pro-angiogenic function of MM-MΦs. **A** Western blot showed the increased expression of MMP2 and VEGF in MM-MΦs derived from both mice and human, relative to MΦs. **B** Western blot showed that knockout of BMI1 impaired the expression of MMP2 and VEGF in MM-MΦs. **C** ELISA assay detected the concentration of VEGF in culture supernatant of murine BM-derived MФs and MM-MФs from wt or BMI1-KO mice. Statistical significance was determined by two-tailed Student *t*-test between wt and BMI1-KO MM-MФs, **P < 0.01. **D** BMI1 knockout lowered the levels of NO in culture supernatant of MM-MФs. Statistical significance was determined by two-tailed Student *t*-test between wt and BMI1-KO MM-MФs, *P < 0.05. **E** GSEA of RNA-seq data showed the enrichment of genes negatively regulating blood vessel endothelial cell migration in BMI1-KO MM-MФs.

TAMs have been shown to promote tumor angiogenesis in many cancers^3,22^. As aforementioned, MM-MΦs had elevated VEGF and NO expression (Fig. 1E, F), both of which mediate angiogenesis in the tumor microenvironment. Western blot analysis revealed elevated VEGF and MMP2^31^ in MM-MΦs (Fig. 5A) but not in BMI1-KO MM-MΦs (Fig. 5B). Additionally, VEGF and NO were also significantly reduced in BMI1-KO MM-MΦs culture supernatant (Fig. 5C, D). Pathway enrichment analysis showed negative modulators of blood vessel endothelial cell migration to be enriched in BMI1-KO MM-MΦs (Fig. 5E). Together, these results suggest that BMI1 elevation promotes angiogenic MM-MΦs.

### MM-MΦs-conferred myeloma chemoresistance requires BMI1 overexpression

**Fig. 6:**
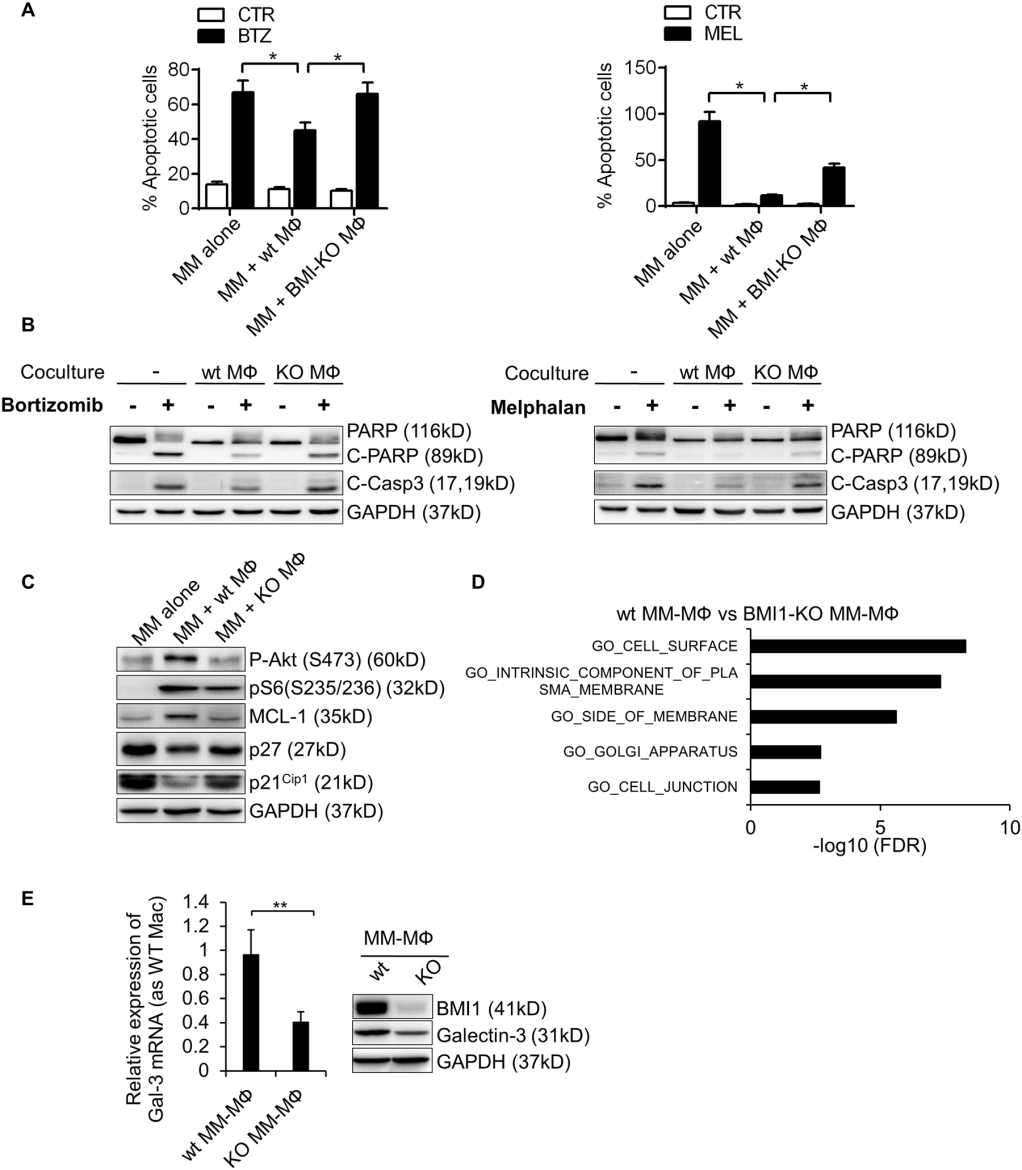
MM-MΦs-conferred myeloma chemoresistance requires BMI1 overexpression. **A** The apoptotic rate of 5TMG1 cells treated with indicated drugs alone or with wt MФs or BMI1-KO MФs for 24 h. Statistical significance was determined by two-tailed Student *t*-test between drug treated 5TGM1 cells cultured alone and with wt MM-MФs, or between drug treated 5TGM1 cells cultured with wt MM-MФs and with BMI1-KO MM-MФs, *P < 0.05. **B** Western blot showed the activation status of apoptotic protein PARP and Capase-3 of 5TGM1 cells treated with indicated drugs alone or with wt or BMI1-KO MФs for 24 h. **C** Western blot showed the protein expression or phosphorylation changes of 5TGM1 cells cultured alone or with wt or BMI1-KO MФs. **D** Pathway enrichment of differential regulated genes from RNA-seq data of wt vs BMI1-KO MM-MФs, comparing with GO cellular component genesets. **E** RT-qPCR showed the mRNA expression of Galectin-3 was decreased in BMI1-KO MM-MФs, relative to wt MM-MΦs (left column). Western blot showed the protein level of Galectin-3 was lower in BMI1-KO MM-MФs (right panel). Statistical significance was determined by two-tailed Student *t*-test between wt and BMI1-KO MM-MФs, **P < 0.01.

We have previously shown that MM-MΦs in the myeloma microenvironment induce drug resistance of myeloma cells^8,9^. To test if BMI1 influences MM-MΦs related chemoresistance, we treated murine MM 5TGM1 cells alone or co-cultured with wt or BMI1-KO MΦs, with bortezomib or melphalan. Apoptosis assays showed that MM cells co-cultured with wt MΦs were most resistant to the drugs, while resistance was suppressed upon BMI1 knockout (Fig. 6A). Western blot showed that wt MΦs-protected MM cells had less PARP and caspase-3 fragmentation, while BMI1-KO MΦs-protected MM cells exhibited significantly higher cell death (Fig. 6B). Analysis of cell survival signaling in MM cells co-cultured with wt or BMI1-KO MΦs showed that MM cells co-cultured with wt MΦs had elevated levels of pAkt, pS6 and antiapoptotic Mcl-1, and reduced levels of the cell cycle inhibitors p21 and p27. The aforementioned cell signaling changes were minor or unchanged in cells co-cultured with BMI1-KO MΦs (Fig. 6C), suggesting that BMI1-KO MΦs do not stimulate MM cell survival signaling. To elucidate BMI1 downstream factors that regulate MM-MΦ-mediated chemoresistance, we used RNA-seq for differential gene expression analysis in wt MM-MΦs vs BMI1-KO MM-MΦs and compared the differential expressed genes with GO term cellular component genesets on MSigDB^28^. This analysis suggested that the cell surface molecule components were impaired in BMI1-KO MM-MΦs (Fig. 6D). Our previous work suggested that MΦ-conferred MM chemoresistance was mediated by cell surface molecule interaction between MΦs and MM cells, thus direct cell contact between the two cell types may be needed to boost MM chemoresistance^8,9^. Therefore, BMI1-KO MM-MΦs may have lost cell surface expression of some key factors, impairing their ability to stimulate MM survival. Of the candidate cell surface genes, we decided to further analyze LGALS3, which encodes Galectin-3 and is reported to modulate TAM functions^32,33^. LGALS3 is also implicated in MM and BM microenvironment interplay^34^. RT-qPCR and western blot analyses revealed significantly reduced Galectin-3 levels in BMI1-KO MM-MΦs (Fig. 6E).

Taken together, these data indicate that BMI1 regulates MM-MΦs-mediated chemoresistance of MM cells. BMI1 upregulation in MM-MΦs is critical for some cell surface molecules expression, including Galectin-3. These surface molecules may mediate crosstalk between MM-MΦs and MM cells, and activate cell survival signaling in MM cells.

### BMI1 regulates MM-MΦ’s pro-myeloma functions in vivo

**Fig. 7:**
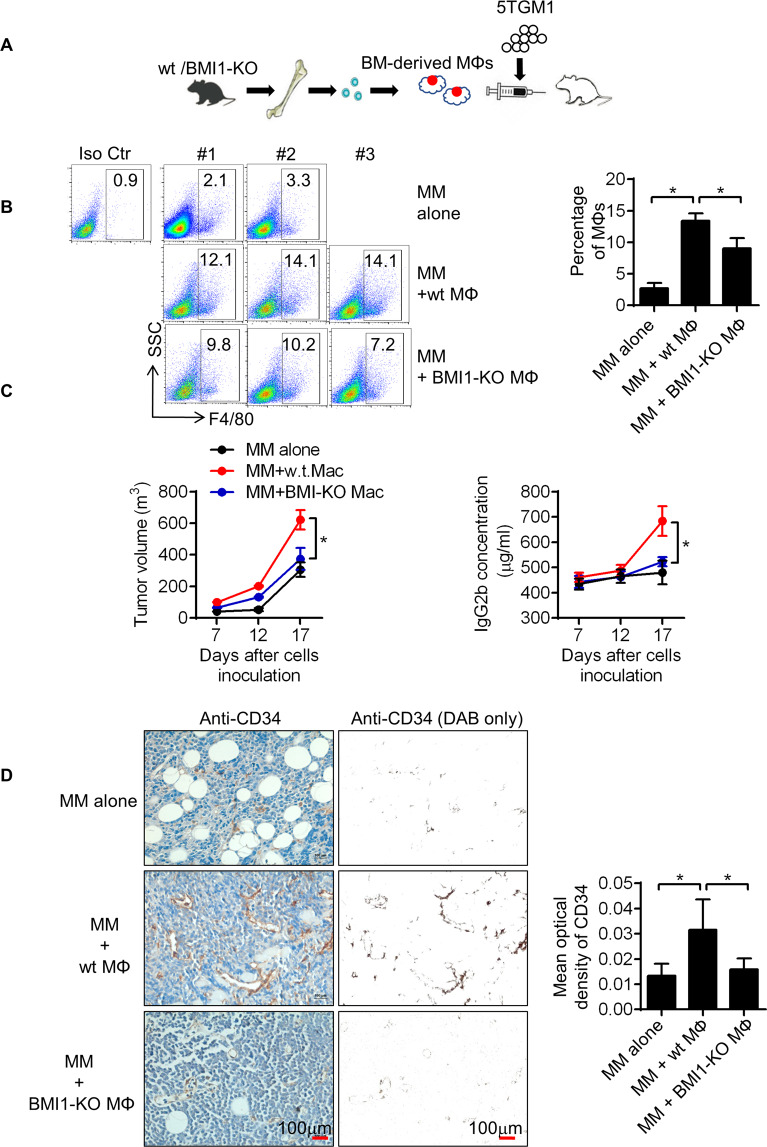
BMI1 regulates MM-MΦ’s pro-myeloma functions in vivo. **A** Schematic displayed that 5TGM1 cells were injected subcutaneously into the B-NDG mice alone or with BM-derived MΦs from wt or BMI1-KO mice. **B** Flow cytometry showed the proportion of MΦs in the subcutaneous tumor beds 7 days after inoculation (Left panel). Tumors with wt MΦs had the most MΦs in tumor beds (right column). Statistical significance was determined by two-tailed Student *t*-test between tumors with 5TGM1 alone and with wt MM-MФs, or between tumors with wt and BMI1-KO MM-MФs, *P < 0.05. **C** Left panel showed the growth curves of 5TGM1 xenografts, wt MΦs were the most potent in promoting tumor growth. Right panel showed monoclonal protein IgG2b concentrations in murine peripheral blood, mice bearing tumors with wt MΦs had the highest concentrations of IgG2b. Statistical significance was determined by two-tailed Student *t*-test between groups with wt and BMI1-KO MM-MФs, *P < 0.05. **D** Representative IHC staining images (left images) and quantification of positive cells (right column) of epithelial cell marker CD34. Statistical significance was determined by two-tailed Student *t*-test between tumors with 5TGM1 alone and with wt MM-MФs, or between tumors with wt and BMI1-KO MM-MФs, *P < 0.05.

To evaluate BMI1 functions in MM-MΦs in vivo, we established a murine MM xenograft model in severely immunodeficient mice (Fig. 7A). MΦs and MM cells were subcutaneously inoculated into the mice and the pro-myeloma functions of BMI1 in MM-MΦs examined. Flow cytometry was used to measure the population of MM-MΦs in the tumor bed 7 days after inoculation. Mice bearing MM with wt MΦs had more MM-MΦs in tumor beds relative to those bearing MM with BMI1-KO MΦs, implying higher proliferation of wt MM-MΦs in vivo (Fig. 7B). Tumor volume and circulating monoclonal protein measurement revealed significantly faster tumor growth in mice bearing MM with wt MM-MΦs relative to mice bearing MM with BMI1-KO MM-MΦs (Fig. 7C). Moreover, relative to BMI1-KO MM-MΦs, MM with wt MM-MΦs tumors exhibited significantly higher levels of CD34, an endothelial cell marker^35^, indicating greater angiogenesis (Fig. 7D). Taken together, these data show that BMI1 drives the pro-myeloma functions of MM-MΦs in vivo.

At last, we examined BMI1 targeting therapy in MM. BMI1 expression has been reported essential for the growth of MM cells^36^. Previous studies showed that BMI1 inhibitors exhibited anti-MM effect in vitro and in vivo^37–39^. We found the BMI1 inhibitor PTC-209 suppressed growth and triggers apoptosis of in vitro cultured MΦs (Supplementary Fig. 4). However, because of its limited drug potency and poor pharmacokinetics, PTC-209 did not enter clinical trials^40^. PTC596 is a second-generation BMI1 inhibitor and has entered four Phase 1 clinical trials (NCT03761095, NCT03605550, NCT03206645, NCT02404480). Based on above evidences, we evaluated the efficacy of PTC596 in MM treatment and MM-MΦ eradication. We found that PTC596 inhibited BMI1 expression in murine BM-derived MΦs and promoted cell death (Supplementary Fig. 5A). In 5 T murine myeloma model, PTC596 administration decreased tumor burden and prolonged mice survival (Supplementary Fig. 5B, C). Upon PTC596 administration, MΦs in myeloma-bearing mice bone marrow were dramatically diminished, while MΦs in healthy mice BM stayed unaffected (Supplementary Fig. 5D, E). The peritoneal MΦs, which were out of the MM BM microenvironment, were not affected by PTC596 in both healthy and myeloma mice (Supplementary Fig. 5F). Overall, our data suggested that BMI1 inhibitors could not only target MM cells, but also eliminate MM-MΦs in treatment of myeloma.

## Discussion

Although MM overall survival has profoundly improved with the introduction of novel agents and immunotherapies, it remains a fatal disease. Currently, MM treatment strategies and ongoing clinical trials focus on eradicating myeloma tumor clones^2^. The BM microenvironment leads to MM treatment failure by mediating plasma cell survival, proliferation, and resistance to chemotherapy^1,41^. Thus, novel strategies that target both myeloma cells and the myeloma microenvironment are needed. The myeloma microenvironment consists of cellular and noncellular compartments^41^. MΦs are major components of the cellular compartment of myeloma BM. Growing evidence indicates that MΦs number in MM tumor bed correlate with MM prognosis. Patients with high BM MΦs infiltration exhibit poorer treatment outcomes and overall survival^42–45^. Wu Y et al. also showed that in addition to MΦ numbers, the polarized differentiation status of MΦs also influences MM prognosis. Patients with M2 MΦs infiltration, characterized by CD163^high^/iNOs^low^ phenotype, have poorer prognosis^45^.

It is not clear how MM-MΦs acquire pro-myeloma function. Here, we find that BMI1 expression in MΦs modulates MM-MΦs’ pro-myeloma features. BMI1 is a member of the Polycomb-group proteins and participates in forming the Polycomb-repressive complex 1 (PRC1), which organizes the chromatin structure to regulate expression of PRC1 target genes^46^. BMI1 has been reported to modulate proliferation of normal and malignant cells^47^. BMI1 function in MM-MΦs has not been determined and few studies have examined BMI1 function in MΦs. BMI1 has been reported to repress IL-10 expression during acute MΦ response to lipopolysaccharide (LPS)^48^. BMI1 has also been reported to suppress pro- and anti-inflammatory MΦs cytokines upon chronic NOD2 stimulation with bacteria-derived muramyl dipeptide (MDP)^49^, implicating BMI1 in M1 MΦ pro-inflammatory responses. Here, we found BMI1 to be upregulated in MM-MΦs. MM-MΦs exhibited pro-myeloma effects in different aspects, including promoting angiogenesis and MM chemoresistance. BMI1 did not seem to be needed for MΦs, as MΦ cell markers were still expressed by BMI-KO MΦs. However, active cell proliferation was lost in BMI-KO MM-MΦs. Importantly, BMI1-KO MM-MΦs lost pro-myeloma features like promoting MM growth, conferring MM drug resistance and angiogenesis in MM tumor bed. Thus, our data suggest that BMI1 is needed for MM-MΦ pro-myeloma activity and that it may be a viable target for reversing MM-MΦ pro-myeloma functions. Currently, anti-TAM (including MM-MΦs) therapeutic strategies include inhibition of TAM recruitment^5^, inhibition of normal MΦs to TAMs transformation^50,51^, direct depletion of MΦs^52,53^, and TAM to normal MΦs reprogramming^54^. It should be noted that BMI1 overexpresses in MM and is becoming an intriguing target in treatment of MM^36,38,39^. In this study, we found that BMI1 inhibitors target MM cells and MM-MΦs, promoting MM treatment. Taken together with our findings that BMI1 upregulation drives MM-MΦ’s pro-myeloma functions, our work provided evidences and molecular basis that the BMI1 inhibitor targeted tumor-promoting MM-MΦs in the MM microenvironment. In future we will explore the interaction of BMI1 overexpressed MM-MΦs with immune cells in myeloma microenvironment and their effects on myeloma tumorigenesis and immune therapy efficacy. Additionally, it may be interesting to evaluate BMI1 expression in TAMs of various human cancers.

In summary, we find that BMI1 is upregulated in MM-MΦs, and that BMI1 modulates MM-MΦ’s pro-myeloma functions. BMI1 inhibitors could not only target MM cells, but also eliminate MM-MΦs in treatment of myeloma. The findings of this study should provide evidences for clinical trials of BMI1 inhibitors in myeloma.

## Materials and methods

### Human specimens

BM aspirates and sections from newly diagnosed MM patients and noncancer donors were provided by the tissue bank, Department of Hematology, West China Hospital, Sichuan University. Ethical approval for the study was granted by the Ethics Committee of West China Hospital, Sichuan University (Protocol No. 114).

### Cell lines

Murine myeloma cell line 5TGM1, 5TGM1-luc (expressing the luciferase gene), human myeloma cell line ARP-1 were kindly provided by Professor Qing Yi (Houston Methodist Cancer Center, Houston Methodist Research Institute, Houston Methodist Hospital, Houston, TX, USA.). Human monocytic leukemia cell line THP-1 was purchased from American Type Culture Collection (ATCC) (VA, USA). All cell lines were cultured in RMPI-1640 (Hyclone, Cat No. SH30809.01, UT, USA) supplemented with 10% FBS (GeminiBio, Cat No. 900-108, CA, USA), at 37 °C, 5% CO2, in a humified incubator. All cell lines were authenticated by short tandem repeat (STR) DNA profiling and tested for mycoplasma contamination before use.

### Drugs and reagents

The BMI1 inhibitor PTC-209 was purchased from Cayman Chemical (MI, USA). The BMI1 inhibitor PTC596, the GLI1/GLI2 inhibitor GANT61, the Smo antagonist Cyclopamine and the c-Myc inhibitor 10058-F4 were purchased from Selleck Chemicals (TX, USA). Recombinant mouse SHH (Cat No. 461-SH-025) was purchased from R&D Systems (MN, USA). Recombinant mouse IL-6 (Cat No. 50136-MNAE) was purchased from SinoBiological (Beijing, China).

### Animals

Mice were housed and maintained in pathogen-free conditions in compliance with requirements from the Animal Care and Use Committees of West China Hospital, Sichuan University. All experimental animal studies were approved by the Animal Care and Use Committees of West China Hospital, Sichuan University.

C57BL/KaLwRijHsd (C57BL/Ka) mice were purchased from Envigo (IN, USA). The 5 T murine myeloma model (5TMM) was generated by tail vein injection of 6-week-old C57BL/Ka mice with 1 × 10^6^ 5TGM1-luc cells. Myeloma was established about 3 weeks after inoculation as determined using IVIS imaging (Caliper Life Sciences, PerkinElmer, MA, USA) and analysis of CD138+ cells in the bone marrow.

We used the 5TMM model to evaluate treatment efficacy of BMI1 inhibitor PTC596 in vivo. Because C57BL/Ka mice and 5TGM1 cells were syngeneic, we reduced the number of animals to five mice per group. Ten 6-week-old C57BL/Ka female mice were intravenously inoculated with 1 × 10^6^ 5TGM1-luc cells, while ten 6-week-old C57BL/Ka female mice without tumor inoculation formed the healthy control group. Mice were randomly divided into four groups, healthy control (without PTC596 treatment, *n* = 5), healthy control (receiving PTC596 treatment, *n* = 5), 5TMM (without PTC596 treatment, *n* = 5), 5TMM (receiving PTC596 treatment, *n* = 5), five mice per group. PTC596 was dissolved in 1% sodium carboxymethyl cellulose and 0.1% Tween-80 and administered by oral gavage at 30 mg/kg per week. Treatment was started 2 weeks after tumor inoculation. Once the mice in 5TMM groups were paraplegia, euthanasia was done for the myeloma mice and the mice in the corresponding healthy control group.

BMI1^fl/fl^ mice (Stock No: 028572), in which Bmi1 exons 4–8 were flanked by loxP sites, were purchased from The Jackson Laboratory (ME, USA). An inducible BMI1-knockout mouse line was generated by crossing the BMI1^fl/fl^ mice with Mx1-Cre C57BL/6 transgenic mice (Fig. 4A). Mice were PCR genotyped. Further details of genotyping primers were given in the Supplementary Materials. To induce BMI1 knockout in adult mice (6–8 week old), BMI1^fl/fl^ and BMI1^fl/fl^Mx1-Cre mice were intraperitoneally injected every other day with five doses (15 mg/kg) of poly I:C (Sigma, Cat No. P1530, MO, USA). 4 weeks after the final poly I:C injection, bone marrow was isolated for in vitro macrophage culture^55,56^.

Severe immunodeficient B-NDG mice (NOD-*Prkdc*^*scid*^ *IL2rg*^*tm1*^/Bcgen) were purchased from Biocytogen (Beijing, China). 5TGM1 cells (1 × 10^6^ cell/mouse) were injected subcutaneously into B-NDG mice, alone or with bone marrow MΦs (5 × 10^5^ cell/mouse) from wild-type or BMI1-KO mice. Because 5TGM1 cells and C57BL/6 mice BM-derived MΦs were syngeneic, we reduced the number of animals to ten mice per group (*n* = 10). Seven days after inoculation, three mice from each group were randomly chosen and euthanatized. The tumors were used for tumor bed MΦs analyses. Tumor volume and monoclonal protein levels were measured on the remaining seven mice from each group. Tumor volume was measured using vernier calipers and calculated as follows: volume = (length × width^2^)/2. Mouse peripheral blood was collected from the angular vein. 5TGM1 monoclonal IgG2b protein levels in the peripheral blood were detected using an ELISA kit (Thermo Fisher Scientific, Cat No. 88-50430-88, MA, USA). All mice were euthanized at the end of the study. Tumors were harvested, fixed, and paraffin embedded for immunohistochemical analyses.

The investigators were blinded to the group allocation when assessing the outcome of animal experiments mentioned above.

### Generation of MΦs and MM-MΦs in vitro

To generate murine BM-derived MΦs, BM cells were collected by crushing the hind leg bones in a mortar in FBS-free RPMI-1640. BM cells were collected by passing the suspension through a cell strainer. Red blood cells were lysed, and cells incubated in FBS-free RPMI-1640 for 2 h. Adherent cells were then cultured in RPMI-1640 supplemented with 10% FBS and 5 ng/mL murine M-CSF (R&D Systems, Cat No. 416-ML) for 7 days into MΦs. The MΦs were then co-cultured with 5TGM1 cells for another 48 h to generate MM-MΦs.

To generate human peripheral blood mononuclear cells-derived MΦs, mononuclear cells from healthy donor peripheral blood were collected through Ficoll density gradient centrifugation. They were then incubated in FBS-free RPMI-1640 for 2 hours and adherent cells cultured in RPMI-1640 supplemented with 10% FBS and 10 ng/mL human M-CSF (R&D Systems, Cat No. 216-MC) for 7 days into MΦs. Human MΦs were co-cultured with ARP-1 cells for another 48 h to generate MM-MΦs. Ethical approval to use human samples was granted by the Ethical Committee of West China Hospital, Sichuan University.

### Protein and mRNA expression analysis

BCA analysis (CWBIO, Cat No. CW0014S, Jiangsu, China) was used to determine protein concentration in cell lysates. Equal protein amounts were boiled in 5× SDS sample buffer (Solarbio, Cat No. P1040, Shanghai, China), resolved by SDS–PAGE, and subjected to western blot analysis with specific primary antibodies. The blots were then incubated with horseradish peroxidase-labeled secondary antibody (Jackson ImmunoResearch Laboratories, PA, USA) and signal developed using enhanced chemiluminescence (Millipore, Cat No. WBKLS0500, MA, USA).

Western blot antibodies against BMI1(Cat No. 5856), c-Myc (Cat No. 13987), pS6 (S235/S236) (Cat No. 4858), pAKT (S473) (Cat No. 4060), PARP (Cat No. 9542), c-Caspase-3 (Cat No. 9661), MCL-1(Cat No. 5453) were purchased from Cell Signaling Technology (MA, USA). Western blot antibodies against VEGF (Cat No.19003-1-AP), MMP2 (Cat No.10373-2-AP), GAPDH (Cat No. 60004-1-Ig) were purchased from Proteintech Group (IL, USA). Western blot antibodies against p21(Cat No. sc-6246), p27(Cat No. sc-1641), Galectin-3 (Cat No. sc-32790) were purchase from Santa Cruz Biotechnology (CA, USA).

Total RNA was extracted using TRI reagent (MRC, Cat No. TR118, OH, USA) and cDNA synthesized using HiScript II Q RT SuperMix (Vazyme, Cat No. R223-01, Nanjing, China) following manufacturer instructions. RT-qPCR analysis was done using the 2× SYBR Green qPCR Master Mix (Bimake, Cat No. B21202, Shanghai, China) following manufacturer instructions. GAPDH was used as an internal control. Further details of qPCR primers were given in the Supplementary Materials.

### Flow cytometry

Apoptosis was analyzed using annexin V staining (Beijing 4 A Biotech, Cat No. FXP023, Beijing, China) following manufacturer instructions. To exclude apoptotic macrophages, myeloma cells were stained with FITC-conjugated CD11b and APC-conjugated annexin V. Apoptotic myeloma cells were CD11b^-^ annexin V^+^.

To examine cell cycle distribution differences between MΦs and MM-MΦs, cells were harvested and fixed with 70% ethanol and stained with 50 µg/mL propidium iodide and100 µg/mL RNase I in PBS for 30 min at 37 °C. Cell cycle distribution was tested by flow cytometry and analyzed by using ModFit software.

Flow cytometry antibodies for human APC-CD163 (Cat No.17-1639-41), PE-Cy7-Ki67 (Cat No. 25-5699-42), and isotype controls were purchased from Thermo Fisher Scientific. Flow cytometry antibodies for human APC/CY7-CD14 (Cat No.301820) and FITC-CD68 (Cat No.333805) were purchased from BioLegend (CA, USA). Flow cytometry antibody for human PE-BMI1(Cat No. 562528) was purchased from BD Biosciences (CA, USA). Flow cytometry antibodies for mouse FITC-CD11b (Cat No. M10117-02E) and MHC-II (Cat No. M100M2-09B) were purchased from Sungene Biotech (Tianjin, China). Flow cytometry antibodies for mouse APC-F4/80 (Cat No.17-4801-82), PE-CD206 (Cat No.12-2061-80) and isotype controls were purchased from Thermo Fisher Scientific.

### CFSE cell proliferation assay

Cell proliferation analysis was done using CellTrace™ CFSE Cell Proliferation Kit (Thermo Fisher Scientific, Cat No. C34554), following manufacturer instructions. MΦs and MM-MΦs were labeled with CellTrace™ CFSE at 1:1000 on Day1 and analyzed by flow cytometry. Cells were then cultured for 5 days and measured the CFSE dilution.

### Detection of nitric oxide

Nitric oxide in the culture supernatant of BM-derived MΦs and MM-MΦs was detected by a Nitric oxide detection kit (Beyotime Biotechnology, Cat No. S0021, Shanghai, China) following manufacturer instructions.

### ChIP-qPCR

Cell nuclei from murine BM-derived MΦs and MM-MΦs, or human PBMC-derived MΦs and MM-MΦs were isolated and chromatin fragmented using a sonicator. Chromatin immunoprecipitation assay (ChIP) was performed using a ChIP assay kit (Millipore, Cat No. 17-611) using an anti-c-Myc antibody (Cell signaling, Cat No. 13987). Precipitated DNA was analyzed by qPCR. Further details of ChIP-qPCR primers were given in the Supplementary Materials.

### Immunofluorescence (IF)

BMI1 expression on CD163 positive cells was determined using IF analysis. Multicolor IF staining of MM patients’ bone marrow paraffin sections was done using the Opal™ four-color manual IHC Kit (PerkinElmer, Cat No. NEL810001KT) following manufacturer instructions. Anti-CD163 antibody (Bio-Rad, Cat No. MCA1853, CA, USA) and anti-BMI1 antibody (Bethyl Laboratories, Inc., Cat No. A301-694A, TX, USA) were used for staining.

### Immunohistochemistry (IHC)

Subcutaneous tumor paraffin sections were deparaffinized, antigen unmasked and stained with anti-CD34 antibody (Beyotime Biotechnology, Cat No. AF1387) at 1:100 dilution in TBST with 1% BSA. Antigen retrieval was done using sodium citrate buffer (10 mM, pH 6.0) at 97 °C for 10 min and cooled to room temperature for 30 min. Sections were then incubated with 3% H_2_O_2_ for 10 min to block endogenous peroxidase activity and then blocked with 5% normal goat serum in TBST for 1 h at room temperature. They were then incubated with anti-CD34 antibody at 4 °C overnight. Biotinylated goat anti-rabbit IgG and streptavidin-biotin complex staining were then performed following manufacturer guidelines (Boster Biological Technology, Cat No. SA1022, China). Signal was then developed using a DAB detection kit (ZSGB-Bio, Cat No. ZLI-9031, China) following manufacturer instructions.

### RNA-seq

Total RNA was extracted using TRI reagent from flow sorted CD11b^+^ F4/80^+^ cells from murine BM-derived MΦs and MM-MΦs. Libraries were prepared using the VAHTS^TM^ total RNA-seq library prep Kit for IIumina (Vazyme, Cat No. NR603). RNA-seq was performed by GENEWIZ (Suzhou, China) on an Illumina Hiseq platform. Raw count RNA-seq data were normalized using the DESeq2 module on the GenePattern environment (https://cloud.genepattern.org). Normalized data were subjected to geneset enrichment analysis (GSEA) using GSEA v4.0.3 program. Hallmark genesets and GO genesets from the molecular signatures database v7.1 were evaluated via GSEA^29^.

### Statistical analyses

All in vitro experiments in this study were repeated three times with similar results obtained. Data were shown as mean ± SD. Statistical analyses were performed on Excel 2013. Two-tailed Student’s *t*-test was used to determine statistical significance between two specific groups (*P*-value). *P* ≤ 0.05 was considered statistically significant.

## Supplementary information

Supplementary Materials

Supplementary figure 1

Supplementary figure 2

Supplementary figure 3

Supplementary figure 4

Supplementary figure 5

Supplementary table 1

## Data Availability

Microarray raw data of human PBMC-derived MΦs and MM-MΦs analyzed during this study are included in this article’s supplementary files. RNA-seq data of murine BM-derived MΦs and MM-MΦs generated and analyzed during the current study are available from the corresponding authors on reasonable request.
