# Supplementary material for: BMI1 regulates multiple myeloma-associated macrophage’s pro-myeloma functions
Source: Cell Death Dis. 2021 May 15;12(5):495. doi: 10.1038/s41419-021-03748-y (PMC8124065; doi:10.1038/s41419-021-03748-y)
Supplement: Supplementary file 1 — Supplementary Materials [file 41419_2021_3748_MOESM1_ESM.doc]

# **Supplementary Materials**

**BMI1 Regulates Multiple Myeloma Associated Macrophage’s Pro-Myeloma Functions**

Danfeng Zhang, Jingcao Huang, Fangfang Wang, Hong Ding, Yushan Cui, Yan Yang, Juan Xu, Hongmei Luo, Yuhan Gao, Ling Pan, Yu Wu, Yuping Gong, Liping Xie, Zhigang Liu, Ying Qu, Li Zhang, Weiping Liu, Wenyan Zhang, Sha Zhao, Qing Yi, Ting Niu, and Yuhuan Zheng

**Supplementary Materials and Methods**

***Mice Genotyping Primers***

Primers used for BMI1 genotying were:

Forward: 5’-CTCAGGAGTAAAATGGACATACCC-3’

Reverse: 5’-TCCATTAGCGTGTAGTAATCCTT-3’

Primers used for Cre genotying were:

Forward: 5’- ATTTGCCTGCATTACCGGTC-3’

Reverse: 5’- ATCAACGTTTTCTTTTCGG-3’

***RT-qPCR Primers***

The following primers were used for RT-qPCR reactions:

Murine BMI1:

Forward: 5’-ATCCCCACTTAATGTGTGTCCT-3’

Reverse: 5’-CTTGCTGGTCTCCAAGTAACG-3’

Murine p16:

Forward: 5’- TGAATCTCCGCGAGGAAAGC-3’

Reverse: 5’-TGCCCATCATCATCACCTGAA-3’

Murine Galcectin-3:

Forward: 5’-CAGAGAGCACTACCCAGGAAAA-3’

Reverse: 5’-TGAGGGTTTGGGTTTCCAGA-3’

Murine GAPDH:

Forward: 5’-AGGTCGGTGTGAACGGATTTG-3’

Reverse: 5’-TGTAGACCATGTAGTTGAGGTCA-3’

***ChIP-qPCR Primers***

Precipitated DNA was analyzed by qPCR using the following primers:

c-Myc binding site at mouse BMI1 promoter region:

Forward: 5’- CCCGACTACACCGACACTAAT-3’

Reverse: 5’- GGCTCCAAAATGGCTCGGA-3’

c-Myc binding site at human BMI1 promoter region:

Forward: 5’-CACGGGCCTGACTACACCGACACT-3’

Reverse: 5’-CACCGCTGAAGGCAGAGTGGAAAC-3’

***Protein synthesis assay***

Protein synthesis levels of MΦs and MM-MΦs were performed using protein synthesis assay kit (Cayman Chemical, Cat No. 601100) following manufacturer protocols.

***THP-1 cell infection and differentiation***

THP-1 cells were infected with control lentivirus and c-Myc expressing lentivirus (purchased from Genechem, Shanghai, China) and selected with puromycin for stable clones. The stable cells were stimulated by 25nM of PMA for 24 hours and rested 3 days in PMA free regular medium (10% FBS 1640 medium) for fully differentiation into MΦs.

**Supplementary Figure legends**

**Supplementary Figure 1**

Protein synthesis assay showed that MM-MΦs had higher protein synthesis than MΦs.

**Supplementary Figure 2**

(A) Western blot showed BMI1 in murine BM-derived MΦs did not change with rmIL-6 treatment. (B) CFSE cell proliferation assay showed GANT61 inhibited proliferation of MM-MΦs. (C) Concentrations of NO in culture supernatant of MM-MФs were decreased with treatment of indicated inhibitors. (D) Western blot showed GANT61 suppressed expression of c-Myc in murine BM-derived MM-MΦs. (E) GANT61 suppressed c-Myc expression of THP-1 derived MΦs, while enforced c-Myc expression overcame this effect. *p<0.05 (Student’s t-test, comparing 2 samples, error bars are SD)

**Supplementary Figure 3**

(A) Flow cytometry showed murine BM-derived MΦs and MM-MΦs from wt or BMI1-KO mice were positive of F4/80 and CD11b expression. (B) Flow cytometry showed the population of MHC-II (M1 MΦ markers) positive cells increased in BMI1-KO MM-MΦs, compared with wt MM-MΦs.

**Supplementary Figure 4**

(A) Bright field photos showed decreased cell number and loss of normal morphology of murine BM-derived MΦs with the treatment of PTC-209 for 24h with indicated concentration. (B) Western blot showed downregulation of BMI1 and cleavage of PARP and Caspase-3 of murine BM-derived MΦs with PTC-209 treatment for 24h with indicated concentration.

**Supplementary Figure 5**

(A) Western blot showed downregulation of BMI1 and cleavage of PARP and Caspase-3 of murine BM-derived MΦs with PTC596 treatment for 24h with indicated concentration. (B) In 5T murine myeloma model, BMI1 inhibitor PTC596 significantly reduced CD138^+^ cell population in bone marrow. (C) Survival curve showed the treatment of PTC596 prolonged the survival of myeloma mice. (D) Flow cytometry showed that PTC596 administration significantly reduced the population of MΦs infiltrating into myeloma bone marrow. Flow cytometry data of 2 representative mice of 5 each group were shown. (E) PTC596 administration significantly reduced BM-infiltrated MΦs in myeloma bearing mice, while PTC596 administration did not change MΦs population in BM of healthy mice. (F) PTC596 administration did not change peritoneal MΦs population of both healthy and myeloma bearing mice. **p<0.01(Student’s t-test, comparing 2 samples, error bars are SD)
