# Supplementary figures and images for: BMI1 regulates multiple myeloma-associated macrophage’s pro-myeloma functions
Source: Cell Death Dis. 2021 May 15;12(5):495. doi: 10.1038/s41419-021-03748-y (PMC8124065; doi:10.1038/s41419-021-03748-y)

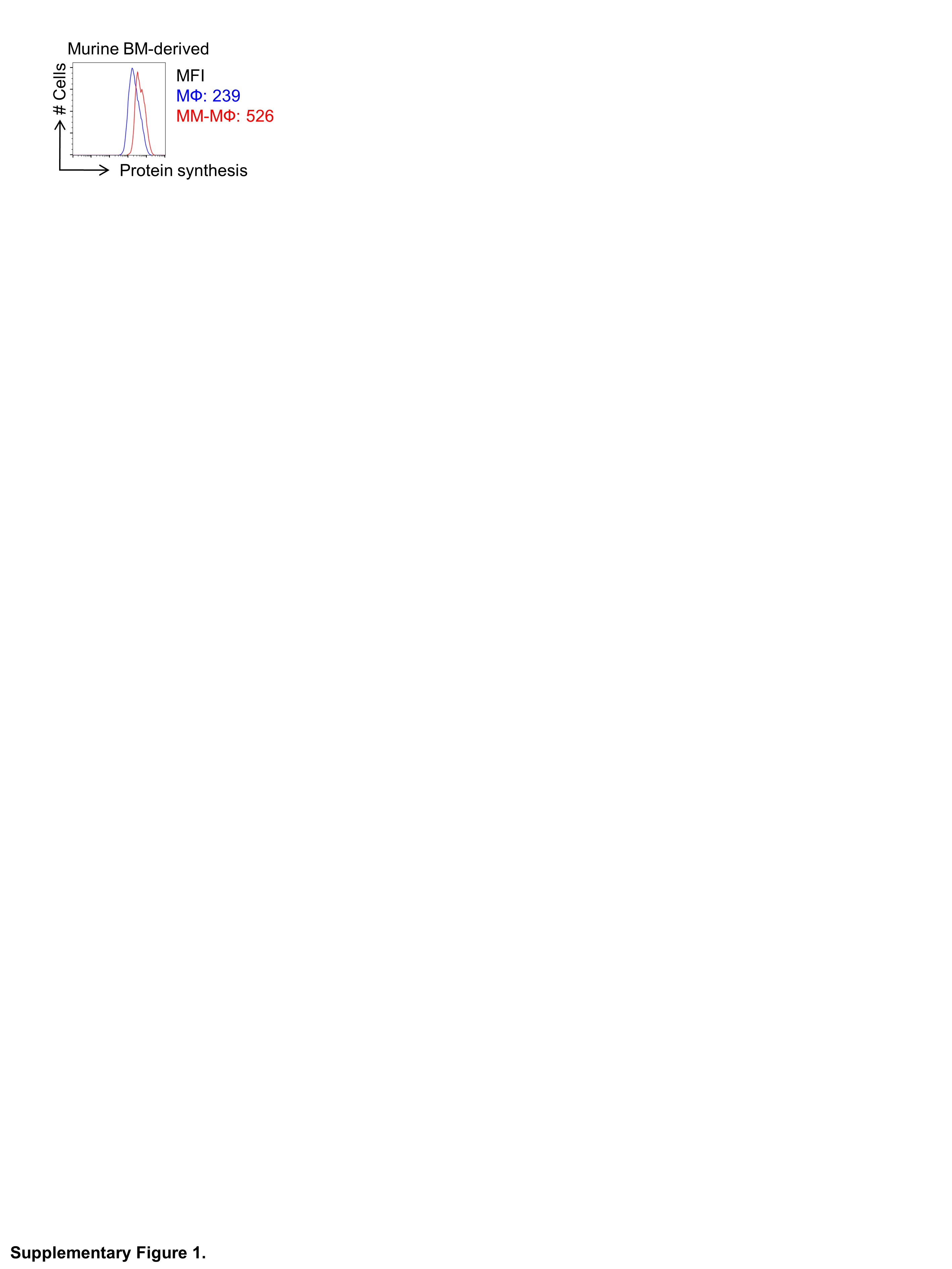

Supplement: Supplementary file 2 — Supplementary figure 1 [file 41419_2021_3748_MOESM2_ESM.tif]

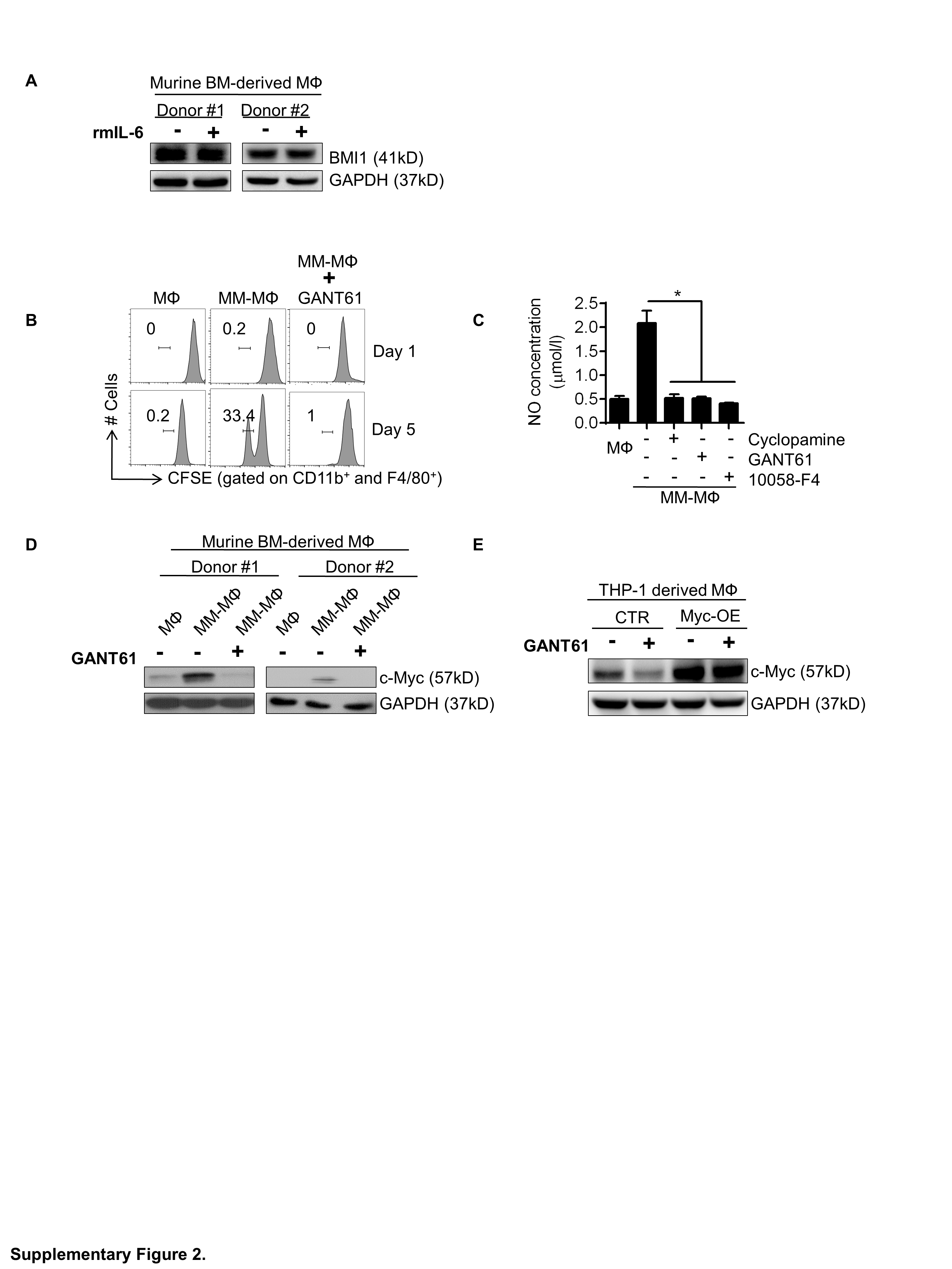

Supplement: Supplementary file 3 — Supplementary figure 2 [file 41419_2021_3748_MOESM3_ESM.tif]

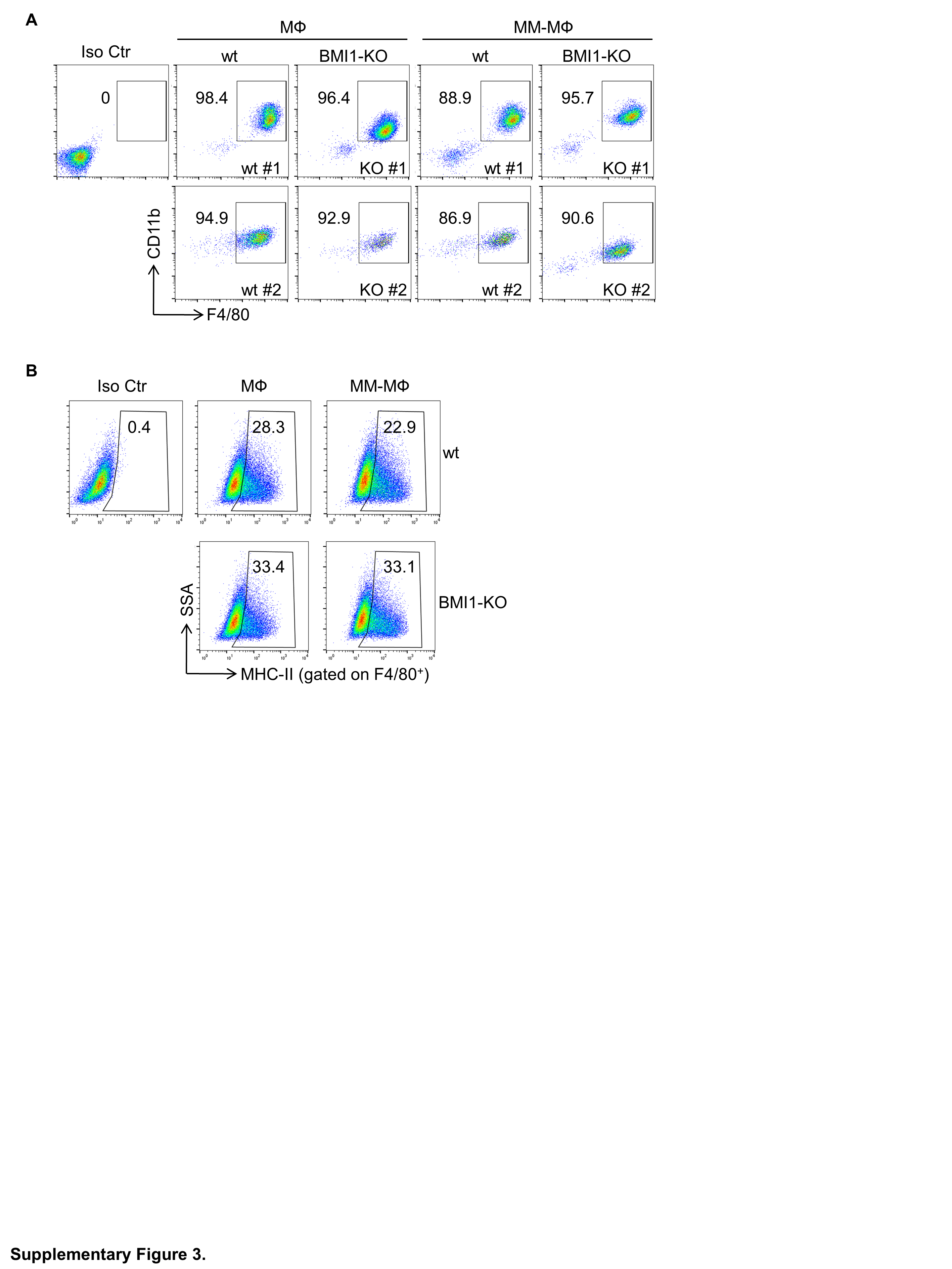

Supplement: Supplementary file 4 — Supplementary figure 3 [file 41419_2021_3748_MOESM4_ESM.tif]

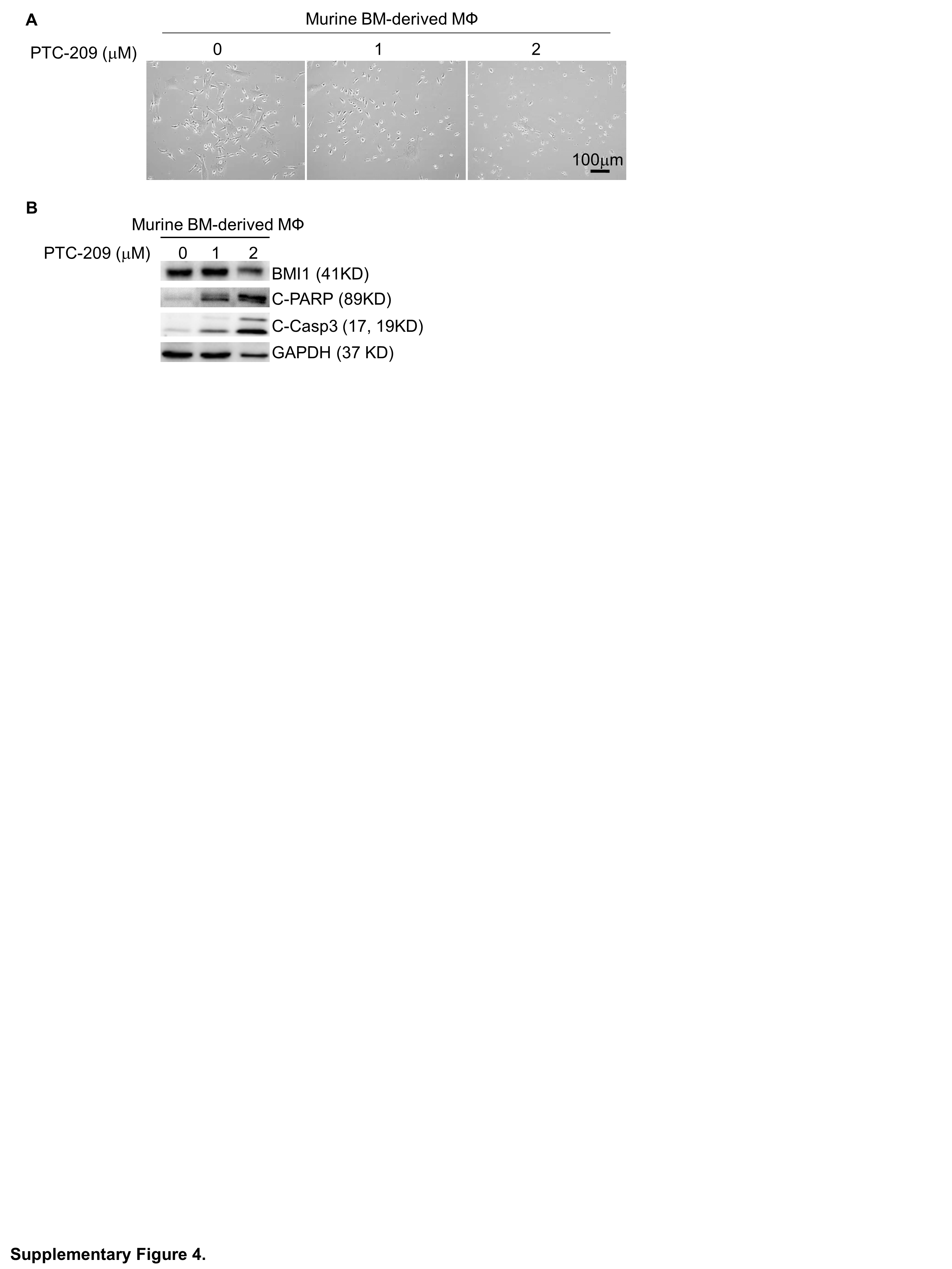

Supplement: Supplementary file 5 — Supplementary figure 4 [file 41419_2021_3748_MOESM5_ESM.tif]

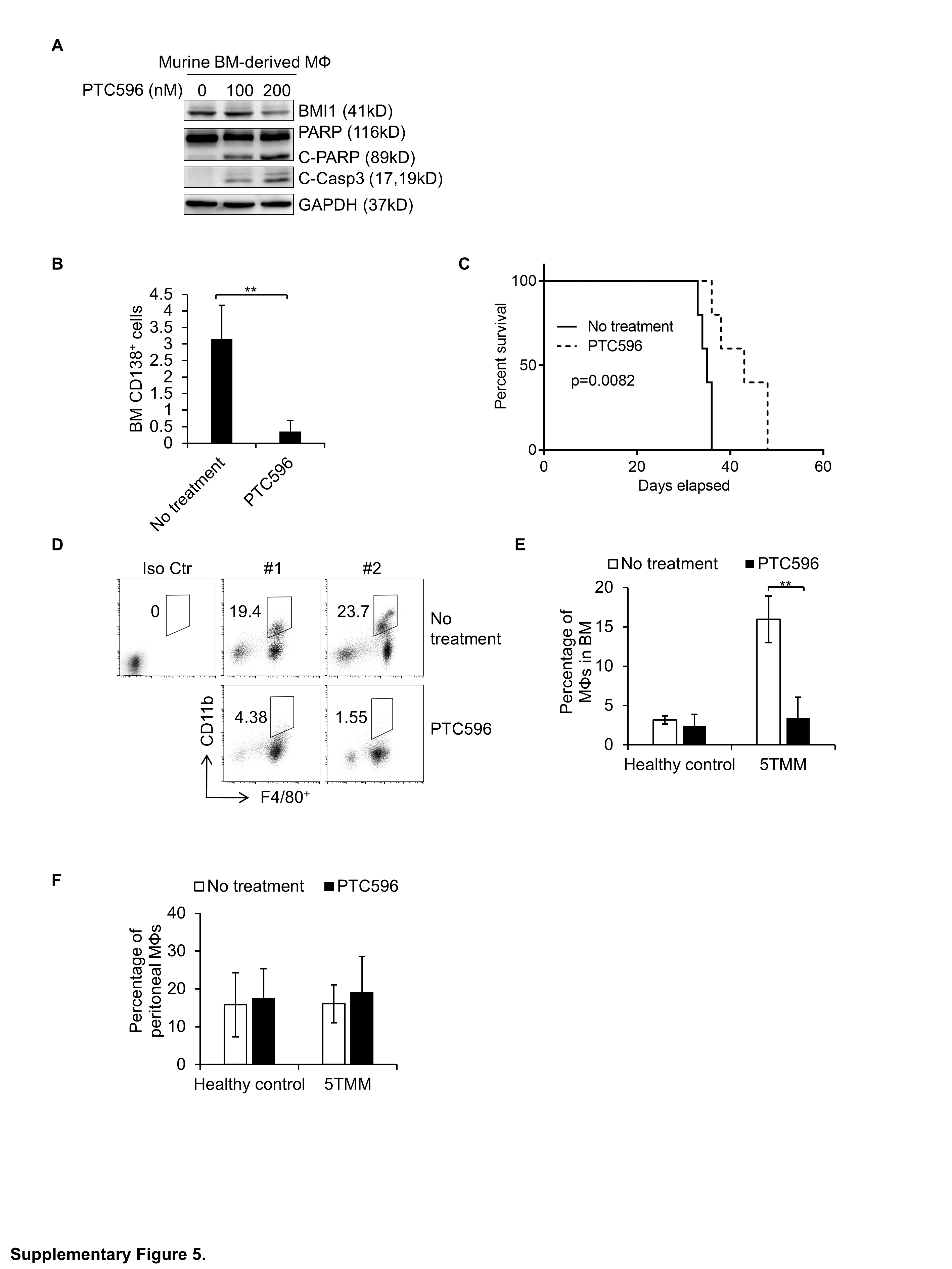

Supplement: Supplementary file 6 — Supplementary figure 5 [file 41419_2021_3748_MOESM6_ESM.tif]
